# Thromboembolic stroke in C57BL/6 mice monitored by 9.4 T MRI using a 1H cryo probe

**DOI:** 10.1186/2040-7378-4-18

**Published:** 2012-09-12

**Authors:** Friederike L Langhauser, Patrick M Heiler, Saskia Grudzenski, Andreas Lemke, Angelika Alonso, Lothar R Schad, Michael G Hennerici, Stephen Meairs, Marc Fatar

**Affiliations:** 1 Department of Neurology, Universitätsmedizin Mannheim, University of Heidelberg, Theodor-Kutzer-Ufer 1-3, 68167, Mannheim, Germany; 2 Computer Assisted Clinical Medicine, Universitätsmedizin Mannheim, University of Heidelberg, Theodor-Kutzer-Ufer 1-3, 68167, Mannheim, Germany; 3 Department of Neurology, University of Würzburg, Josef-Schneider-Str. 11, 97080, Würzburg, Germany

**Keywords:** Embolic stroke, Experimental, T-PA, MRI, Animal models

## Abstract

**Background:**

A new thromboembolic animal model showed beneficial effects of t-PA with an infarct volume reduction of 36.8% in swiss mice. Because knock-out animal experiments for stroke frequently used C57BL76 mice we evaluated t-PA effects in this mouse strain and measured infarct volume and vascular recanalisation *in-vivo* by using high-field 9.4 T MRI and a ^1^H surface cryo coil.

**Methods:**

Clot formation was triggered by microinjection of murine thrombin into the right middle cerebral artery (MCA). Animals (n = 28) were treated with 10 mg/kg, 5 mg/kg or no tissue plasminogen activator (t-PA) 40 min after MCA occlusion. For MR-imaging a Bruker 9.4 T animal system with a ^1^H surface cryo probe was used and a T2-weighted RARE sequence, a diffusion weighted multishot EPI sequence and a 3D flow-compensated gradient echo TOF angiography were performed.

**Results:**

The infarct volume in animals treated with t-PA was significantly reduced (0.67 ± 1.38 mm^3^ for 10 mg/kg and 10.9 ± 8.79 mm^3^ for 5 mg/kg vs. 19.76 ± 2.72 mm^3^ ; p < 0.001) compared to untreated mice. An additional group was reperfused with t-PA inside the MRI. Already ten minutes after beginning of t-PA treatment, reperfusion flow was re-established in the right MCA. However, signal intensity was lower than in the contralateral MCA. This reduction in cerebral blood flow was attenuated during the first 60 minutes after reperfusion. 24 h after MCA occlusion and reperfusion, no difference in signal intensity of the contralateral and ipsilateral MCAs was observed.

**Conclusions:**

We confirm a t-Pa effect using this stroke model in the C57BL76 mouse strain and demonstrate a chronological sequence MRI imaging after t-PA using a ^1^H surface cryo coil in a 9.4 T MRI. This setting will allow testing of new thrombolytic strategies for stroke treatment *in-vivo* in C57BL76 knock-out mice.

## Background

Approximately 75% of all stroke subtypes are caused by embolism leading to cerebral vessel occlusion [1]. The only approved specific treatment for acute stroke is thrombolysis with tissue plasminogen activator (t-PA), which restores cerebral blood flow and improves the neurological outcome in patients with acute ischaemic stroke. This therapy however is associated with certain risks, such as secondary haemorrhage after stroke. New treatment strategies are therefore required, rendering the development of new animal models in combination with adequate imaging strategies. Orset and colleagues [2] demonstrated a stroke model in mice with t-PA induced reperfusion mimicking the clinical situation. In this model thromboembolic stroke is induced by local injection of purified thrombin directly into the right MCA. The resulting fibrin-rich embolus can be completely dissolved by t-PA [2].

The *in vivo* localisation and classification of stroke in the murine brain model and the determination of the lesion size by means of magnetic resonance imaging (MRI) is still challenging, because very small anatomical sizes require ultra high spatial resolution. The signal-to-noise ratio (SNR) increase of a cryogenic transmit/receive coil compared to a room temperature coil of comparable dimensions by a factor of 2.5 makes imaging of the murine brain at an adequate spatial resolution possible without the use of any exogenous contrast agents [3,4].

The purpose of this study was to evaluate this thromboembolic model in C57BL/6 mice and to determine the infarcted area after MCA occlusion (MCAo) with and without subsequent reperfusion by means of MRI. Furthermore, a common three-dimensional time of flight (3D-TOF) angiography [5-7] was used with the cryo coil to demonstrate time development of the t-PA induced reperfusion.

## Methods

### Animals

Male C57BL/6 mice (25–30 g; Charles River, Sulzfeld, Germany) – not swiss mice as in Orset et al [2]- were housed under standard conditions with open access to food and water. All experiments were approved by the local Ethical Committee in accordance with the animal protection guidelines and European Communities Council guidelines.

### MCA occlusion

The animals were anesthetised with 1.5-2% isofluran (Abbott, Wiesbaden, Germany) in a mixture of oxygen/air (1:1) administered via a face mask and placed in a stereotactic frame. MCA occlusion was induced by thrombin injection, as described elsewhere [2]. Briefly, the skin between the right eye and the right ear was incised and the temporal muscle was retracted. To expose the MCA, a small craniotomy was performed and the dura mater was excised. A micropipette was introduced into the lumen of the MCA and 2 μL (1.5 U) of purified murine alpha-thrombin (Haematologic Technologies, Vermont, USA) were injected to induce the formation of a clot. Ten minutes after injection the clot had stabilised and the micropipette was removed. After surgery the incision wound was sutured.

### T-PA induced thrombolysis

In 20 animals t-PA (Actilyse, Boehringer Ingelheim, Germany) was intravenously injected into the tail vein 40 min after MCAo to induce thrombolysis. This was not in accordance with the described model by Orset and colleagues [2], as the thrombin formerly used was no longer available by the manufacturer and our previous (unpublished) results indicate that a longer clotting time is needed to obtain reliable results (40 min vs. 20 min used by Orset et al [2]). One group (n = 12, group 1) received 5 mg/kg t-PA and a second group (n = 8, group 2) 10 mg/kg t-PA. 10% of t-PA was administered as a bolus and 90% as a continuous infusion in a total volume of 250 μL over a 40 min interval using a syringe infusion pump (CMA, Stockholm, Sweden). Control animals (n = 8, group 3) were infused with 250 μL saline under the same conditions.

Three animals (group 4) received 10 mg/kg t-PA 90 min after MCAo inside the MRI for chronological sequence imaging of reperfusion. All animals were sacrificed after 1 week.

### Functional testing

Functional scores adapted from Chen et al [8] were obtained one day after stroke induction and before sacrifice.

### MRI

The imaging experiments of groups 1–3 were performed on a 9.4 T Biospec 94/20 USR (Bruker, Germany) small animal system equipped with 740 mT/m gradients and a ^1^H surface cryo probe (Bruker, Germany) 24 h after MCAo (and t-PA administration respectively). The animals were anesthetised with 1.5-2% isofluran and positioned into the magnet with a laser-controlled system for the animal cradles. Respiratory frequency and body temperature were monitored throughout the experiment and the latter was maintained with a water heating pad. The protocol consisted of a T2-weighted RARE sequence [9], a diffusion weighted multi-shot EPI sequence to obtain the apparent diffusion coefficient (ADC)-maps [10] and a 3D flow-compensated gradient echo TOF angiography [5,6]. Sequence parameters were set as follows. RARE: TR = 2.5 s; effective TE = 60 ms; echo train length = 4; 4 averages; matrix size = 384 × 384; FOV = 17 × 17 mm^2^ ; slice thickness = 0.4 mm; measurement time (12 slices) = 6 min 40 s. EPI-Diffusion: 4 phase encoding steps, TR = 3 s; TE = 20 ms; 4 averages; matrix size = 128 × 128; slice thickness = 0.4 mm; 3 orthogonal diffusion directions with three b-values b = 0, 100 s/mm^2^ , 1000 s/mm^2^ ; measurement time (12 slices) = 5 min 36 s. 3D-TOF: TR = 22 ms; TE = 3.9 ms; Flip angle = 40°; matrix size: 256 × 256 × 128; FOV = 16 × 16 × 16 mm^3^; measurement time = 15 min 46 s. Angiograms were obtained by generating maximum intensity projections (MIPs) using ImageJ software (National Institutes of Health, Bethesda, MD). With the experimental setup and the described protocol it is possible to acquire T2- weighted images, ADC maps and TOF-MIPs within a measurement time of 28 min.

### Histology

Seven days after MCAo – in contrast to 24 h in the model by Orset et al [2] - the animals were re-anesthetised as described above and perfused with 50 mL of 4% formaldehyde in PBS. Their brains were removed, formalin-fixed and paraffin-embedded. Serial coronal sections (3 μm) were cut using a RM2265 microtome (Leica, Bensheim, Germany) and mounted on silane-coated glass slides. Every 200 μm one slice was stained with haematoxylin-eosin to visualise the infarctions.

### Assessment of infarct volumes and statistical analysis

Planimetric measurements of MRI images (ImageJ software, National Institutes of Health, Bethesda, Md) were performed and used to calculate lesion volumes, which were corrected for brain oedema as described [11].

Data are expressed as mean ± standard deviation and were analysed statistically using the PrismGraph 4.0 software package (GraphPad Software). Data were analysed by Bonferroni-corrected one-way ANOVA. P-values less than 0.05 were considered to be statistically significant.

## Results

### Infarct volume

**Figure 1:**
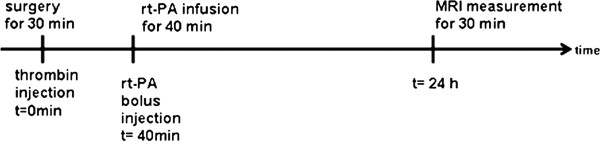
Experimental timeline of MCA occlusion and reperfusion.

**Figure 2:**
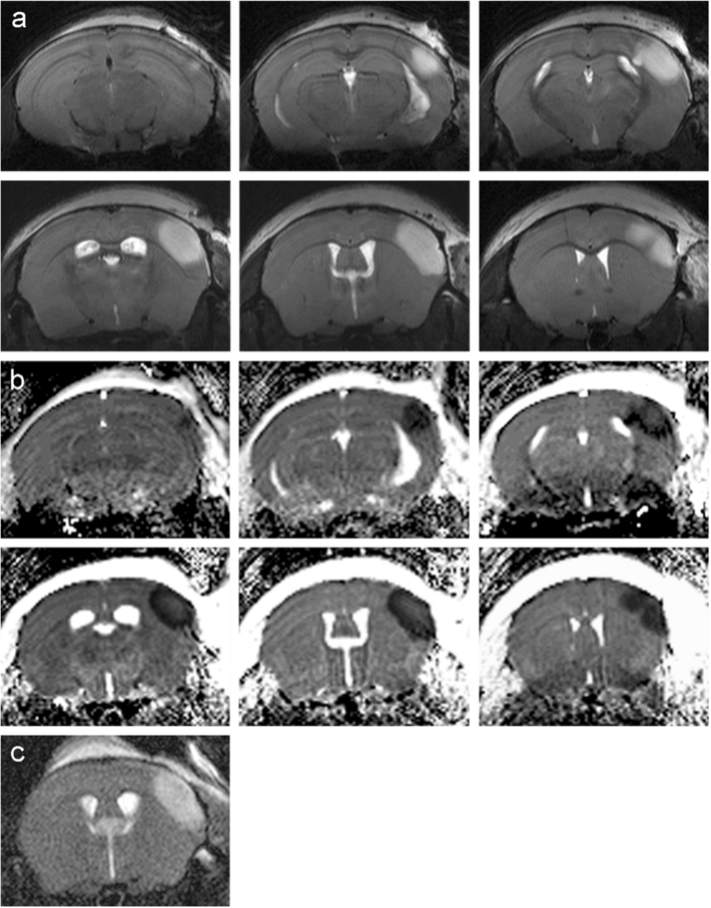
**T2-weighted RARE images using the cryo-probe of consecutive slices acquired 24 h after MCAo clearly demonstrate the infarcted region **(**a**)**. **ADC maps calculated of the diffusion-weighted images showing a significant decrease of the ADC in the ischaemic region (**b**). Slice positioning is identical to Figure 2a. T2-weighted RARE image of the same mouse using a ^1^H linear birdcage resonator (72 mm i.d.) instead of the cryo-probe (**c**).

Figure 1 illustrates the experimental protocol with thrombin injection, t-PA administration and MRI after 24 h. The infarcted region appears hyperintense in the T2-weighted images (Figure 2a). Because of absence of ischemic lesion on MRI we excluded five animals in the 10 mg group, two in the 5 mg group and two in the saline group before further analysis. Saline treated animals showed ischemic lesions that were restricted to the cortex with a mean infarct volume of 19.8 ± 2.7 mm^3^ (n = 8). The ADC maps (Figure 2b) calculated of the diffusion-weighted images revealed a significant signal decrease in the region corresponding to the T2 hyperintense area.

### Reduction of infarct volume by t-PA infusion

**Figure 3:**
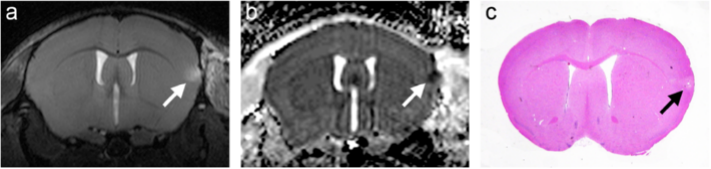
**Turbo RARE images of consecutive slices acquired after MCAo and subsequent administration of t-PA possess only small residual infarcted regions **(**a**)**. **ADC maps show a signal decrease in the identical area (**b**). On the haematoxylin-eosin stained slice the infarcted region is consistent with the MR images (**c**).

**Figure 4:**
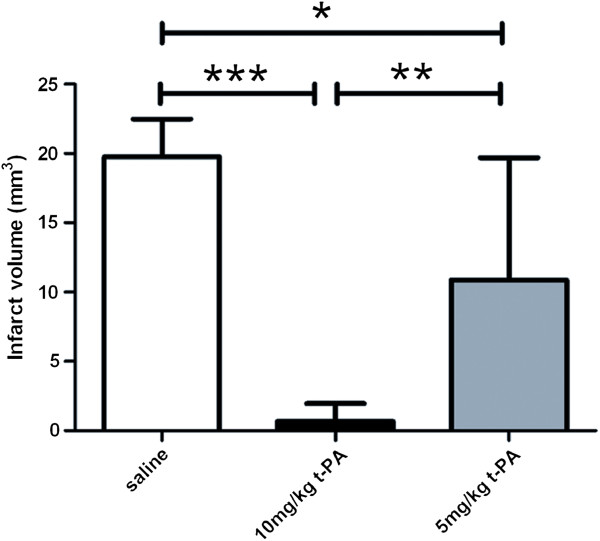
Volume of the ischemic lesion of controls (white bar) and t-PA treated mice with 10 mg/kg (black bar) and 5 mg/kg (grey bar) quantified by MRI 9.4 T - T2 images. ***, p < 0.0001, **, p < 0.001 and *, p < 0.05, one-way ANOVA, Bonferroni post-hoc test.

Twenty animals were intravenously injected with t-PA 40 min after clot formation. Figure 3 shows one example of the MRI measurements, 24 h after MCAo and therapy with 10 mg/kg t-PA and its corresponding HE stained coronary brain slice. Treatment with t-PA led to a significant reduction of the infarct volume. The damaged area calculated from the T2-weighted images is 0.7 ± 1.3 mm^3^ (n = 8). This represents a reduction of the infarct size by approximately 96% (Figure 4). In the haematoxylin-eosin stained brain sections as well, only a small residual infarcted region is apparent (Figure 3c). By using 5 mg/kg of t-PA we found an infarct volume between the group of 10 mg/kg and saline, with 10.9 ± 8.8 mm^3^ (n = 12).

### Time course of reperfusion

**Figure 5:**
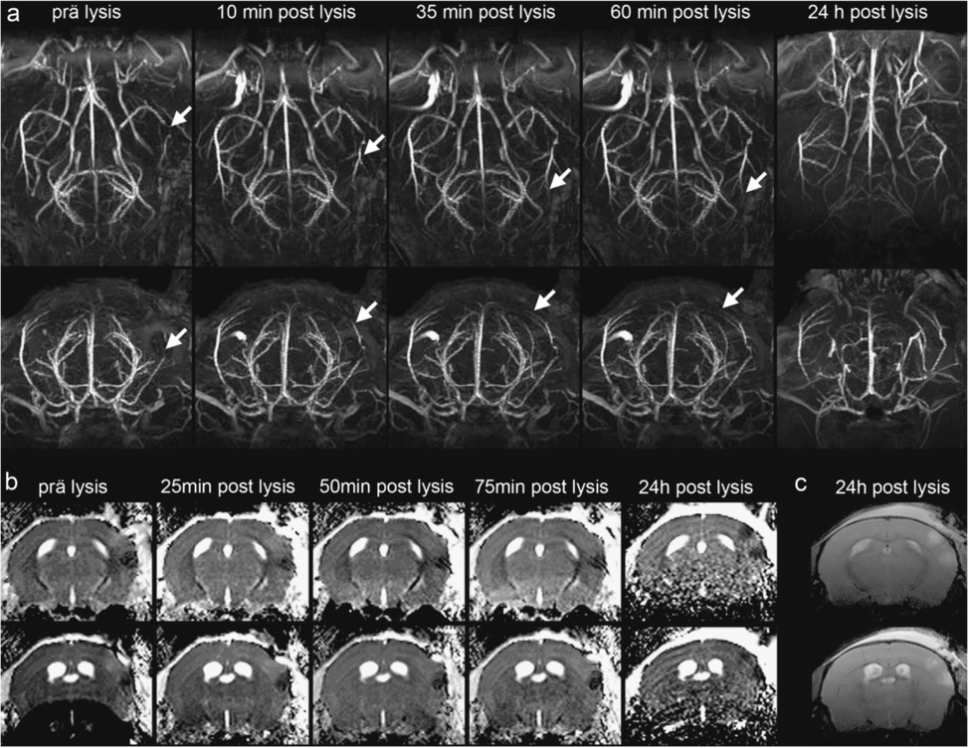
**High resolution MR angiography of a mouse during reperfusion. **Before t-PA treatment, there is no cerebral blood flow in the right MCA. Ten minutes after starting t-PA infusion, flow on the MCA is re-established. However, signal intensity is lower than in the contralateral MCA. This reduction in cerebral blood flow is attenuated during the first 60 min after reperfusion. 24 h after MCAo and reperfusion no difference in signal intensity of the contralateral and ipsilateral MCAs is obvious (**a**). ADC Maps during reperfusion (**b**). In the first 75 min after reperfusion a slight decrease in the signal intensity of ADC is visible and 24 h after reperfusion the infarcted area is larger than at the onset. This is also detectable in the T2-weighted measurement (**c**).

Three animals (group 4) were reperfused with t-PA after clot formation inside the MRI for chronological sequence imaging of reperfusion. Figure 5a demonstrates serial maximum intensity projections (MIP) of the 3D-TOF measurement along the coronal (upper row) and axial (lower row) direction taken during reperfusion. Prior to t-PA bolus injection there was no visible cerebral blood flow in the right MCA posterior to the occlusion (arrow). Already in the first scan ten minutes after bolus injection, blood flow on the right MCA was apparent. However, signal intensity was lower than on the contralateral MCA. This reduction in cerebral blood flow was attenuated during the first 60 min after reperfusion. 24 h after MCAo and reperfusion, no difference in the signal intensities of the contralateral and ipsilateral MCAs was visible.

In the ADC maps there was a minor reduction in the volume of ADC after t-PA bolus injection (Figure 5b). In the following 22 h after reperfusion the ADC hypointense area increased. No hyperintense signal in the T2-weighted images could be seen in the first two hours after stroke induction (data not shown). 24 h after clot formation the infarcted area in the T2-weighted images (Figure 5c) was consistent with the area seen in the ADC maps.

### Functional testing

Neurological deficits were measured one day after MCAo using the modified Neurological Severity Score (mNSS) [8] which is a composite of motor, sensory, reflex and balance tests. We could not observe any neurological deficits neither in mice in the saline treated nor in the t-PA treated group one day after surgery or before sacrifice by using this score system.

## Discussion

In this study we demonstrate that MRI at high magnetic field strength with 9.4 T in combination with the use of a cryo probe facilitates efficient stroke imaging even in the mouse brain with very small anatomical sizes. These measurements allow an accurate determination of the infarcted area and even reperfusion *in vivo* and validate the variation of the infarcted area in a time-dependent manner in a mouse model of thromboembolic stroke.

We used the protocol for stroke induction referring to the work from Orset et al [2]. The advantage of this model compared to other embolic stroke models [12,13] is the high reproducibility. The clot is always located at the same place and so far no secondary microthrombi in small arteries or capillaries could be observed [2]. Therefore this model leads to clot formation with accurate and reproducible brain damage.

As many knockout mice are bred in a C57BL/6 background we adapted the protocol established in swiss mice for the C57BL/6 mouse strain. It is known that the susceptibility to cerebral ischemia can vary in different mouse strains [14]. One indication for the varying susceptibility is that we observed greater infarct volumes in non-t-PA treated animals compared to Orset et al. [2] and had to use a higher amount of thrombin (1.5U compared to 0.75U) to get reliable infarcts. In contrast the infarct volume in t-PA treated animals was smaller than in the work by Orset et al. [2] despite later injection - after 40 min post-stroke and not after 20 min. The efficacy of t-PA treatment with 10 mg/kg body weight was quite high, reducing the infarct volume below 10 mm^3^. We therefore suggest using the lower tPA concentration (5 mg/KG) for evaluating combination therapies with tPA in further studies if using this animal strain and this model. By using 5 mg/kg body weight of t-PA we found a favourable dose dependency concerning the infarct volume compared to 10 mg/kg t-PA and saline treatment.

However, for adjunct therapies of t-PA this model is superior to others because of its local thrombus formation, the dose-dependent therapeutical effect of t-PA and the opportunity of giving additional medical treatment. The non-invasive proof of infarction, reperfusion and temporal evolution could be easily evaluated by our presented MRI technique using a cryo probe. The advantage of using this high-field MR technique could be demonstrated in Figure 5b where a hyperintense region beside the hypointense lesion could be seen in ADC images before thrombolysis. This hyperintensity diseappers in the early recanalisation period (50 and 75 min post t-Pa) but reappear 24 h later as a second ischemic lesion, probably representing a reperfusion injury. Beckmann and colleagues [7] used a 3D gradient-echo sequence to visualize the cerebral vasculature in the mouse brain in a model of cerebral ischemia. They used a mouse model of focal cerebral ischemia where a thread is inserted into the internal carotid artery to occlude the origin of the MCA. The mouse model we used with local injection of purified thrombin directly into the right MCA followed with t-PA induced reperfusion, simulates the clinical situation more realistically than the filament model. Furthermore the 3D-TOF measurements used in this study can visualize single blood vessels with a small diameter of approximately 100 μm and deliver a proper and direct assessment of the success of occlusion of the murine MCA and enables the visualization of reperfusion at different time points even in distal MCA branches. Furthermore, it is possible to depict the development of t-PA induced reperfusion as a function of time. To proof the occlusion and reperfusion by MRI measurements provides more reliable data than measuring the local cerebral blood flow by laserdoppler flowmetry as used in the work of Orset et al [2], because the MRI measurement is not as susceptible to extern influences as the laserdoppler probe. Another method for evaluation of cerebral blood flow is continuous arterial spin labeling (CASL), which will be even better if a cryo coil – like in our experiments - is used [7].

Another important advantage of this animal model is the low mortality rate of the infarcted mice. In other stroke models the number of animals that die during the surgical procedure or in the time until evaluation of brain damage is quite high [15,16]. No of the 28 animals used in this study died during surgery or in the first seven days. This makes evaluation of long term outcome like inflammatory processes, neuroplasticity or neuroregeneration possible.

In summary, the use of a cryo probe has improved the imaging quality tremendously. The opportunity to view recanalisation by MR-angiography allows therapeutical interventions in a model of clot and clot-lysis and therefore extends the list of possible treatments and applications in this stroke animal model and C57BL76 mice.

## Competing interests

The authors declare that they have no competing interests.

## Authors´ contributions

MF directed the study, designed experiments, analyzed data and drafted the manuscript; FL, designed and performed experiments, analyzed data and drafted the manuscript; PH, SG, AL and AA designed and performed experiments, analyzed data and contributed to manuscript writing; LRS provided specific input to MRI experiments including experimental design and data analysis; MGH, SM funded the study, and designed experiments. All authors read and approved the final manuscript.
